# Supplementary material for: Density-Controlled Growth of ZnO Nanowalls for High-Performance Photocatalysts
Source: Materials (Basel). 2022 Dec 16;15(24):9008. doi: 10.3390/ma15249008 (PMC9780911; doi:10.3390/ma15249008)
Supplement: Supplementary file 1 [file materials-15-09008-s001.zip › materials-2051109 - supplementary.pdf]

materials

Supplementary Materials

Density-Controlled Growth of ZnO Nanowalls for High-Performance Photocatalysts

Yu-Cheng Chang [1,*], Ying-Ru Lin [1,2], Sheng-Wen Chen [1] and Chia-Man Chou [3,4,5,*]

1 Department of Materials Science and Engineering, Feng Chia University, Taichung 407102, Taiwan
2 Department of Materials Science and Engineering, National Yang Ming Chiao Tung University, Hsinchu 30010, Taiwan
3 Department of Surgery, Taichung Veterans General Hospital, Taichung 40705, Taiwan
4 College of Medicine, National Yang Ming Chiao Tung University, Taipei 11221, Taiwan
5 Department of Post-Baccalaureate Medicine, National Chung Hsing University, Taichung 40227, Taiwan
* Correspondence: yuchchang@fcu.edu.tw (Y.-C.C.); cmchou@vghtc.gov.tw (C.-M.C.)

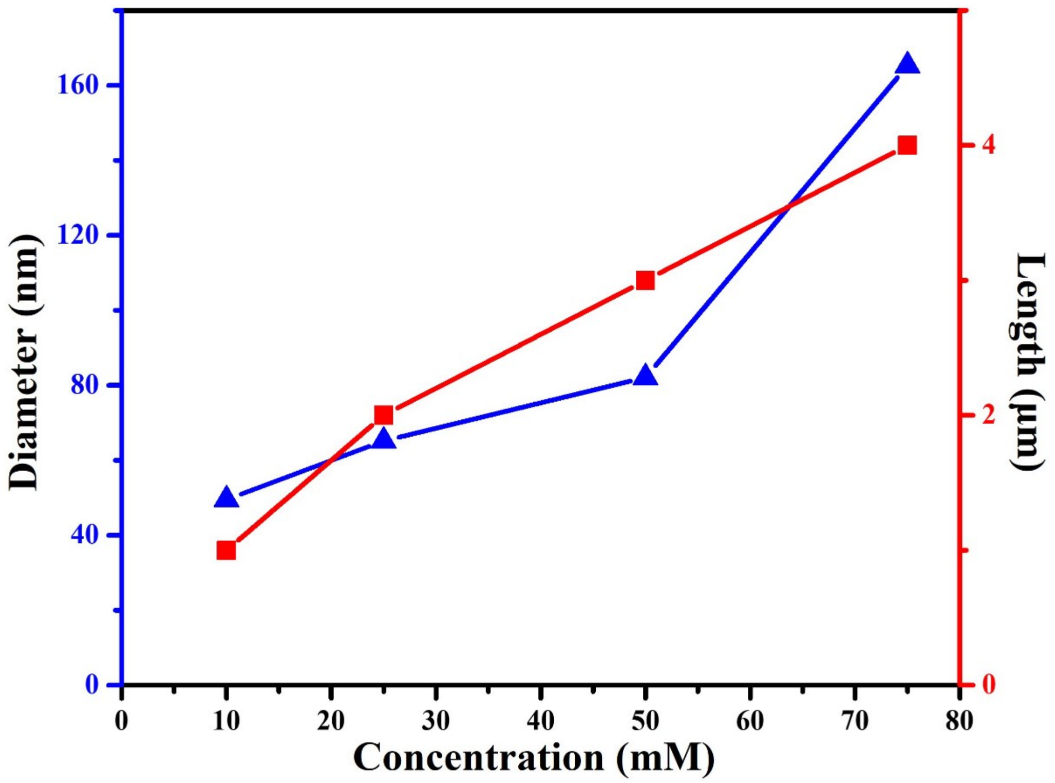

Figure S1. The diameter and length of ZnO nanowires were plotted as a function of the different concentrations of ZnO precursors.

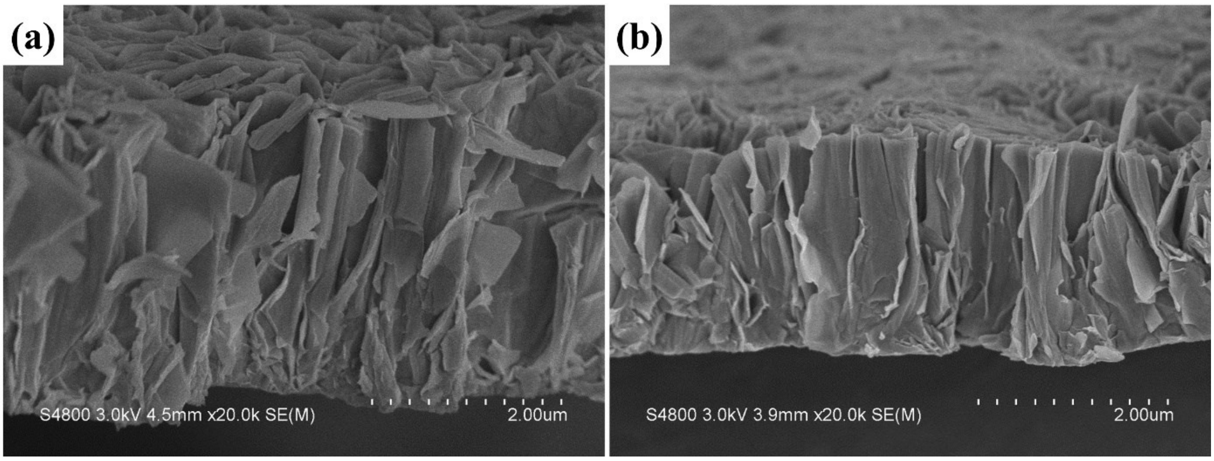

Figure S2. The cross-sectional FESEM images of the ZnO nanowalls synthesized at the different concentrations of ZnO precursors. The concentrations of ZnO precursors are (**a**) 50 and (**b**) 75 mM, respectively.
